# Topological Analysis of Functions on Arbitrary Grids: Applications to Quantum Chemistry

**DOI:** 10.1021/acs.jctc.2c00649

**Published:** 2022-09-07

**Authors:** Michael J. Hutcheon, Andrew M. Teale

**Affiliations:** School of Chemistry, University of Nottingham, University Park, Nottingham NG7 2RD, U.K.

## Abstract

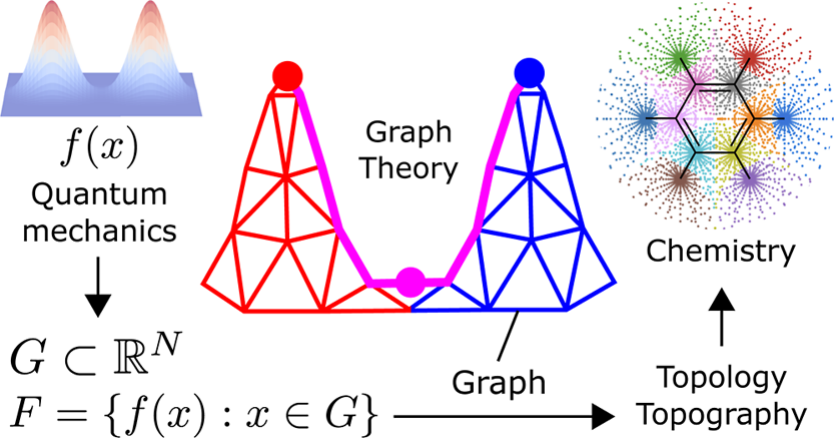

Algorithms are presented for performing a topological analysis of an arbitrary function, evaluated on an arbitrary grid of points. These algorithms work strictly by post-processing the data and require no additional function evaluations. This is achieved by connecting the grid points with a neighborhood graph, allowing the topological analysis to be recast as a problem in the graph theory. The flexibility of the approach is demonstrated for various applications involving analysis of the charge and magnetically induced current densities in molecules, where features of the neighborhood graph are found to correspond to chemically relevant topographical properties, such as Bader charges. These properties converge using orders of magnitude fewer grid points than uniform-grid approaches while exhibiting an appealing *O*[N log(*N*)] scaling of the computational cost. The issue of grid bias is discussed in the context of graph-based algorithms and strategies for avoiding this bias are presented. Python implementations of the algorithms are provided.

## 1 Introduction

First-principles quantum mechanical calculations have been widely successful in describing chemical processes at a fundamental level. However, the interpretability of these calculations is still an ongoing subject of debate.^2,3^ How does one move between the electrons and nuclei of first-principles calculations to the more intuitive building blocks of chemistry, such as atoms, bonds, lone-pairs, and so forth? Significant progress has been made in the forward direction by considering the topography and topology of quantum-mechanical objects in a field that has become known as quantum-chemical topology (QCT).^4−6^ Early examples, such as Bader’s partitioning of atoms-in-molecules (AIM) via the basins of attraction of the electron density, ρ, demonstrated that it was possible to recover the concepts of atoms^7^ and bonds.^8^ Later, such methods were generalized and applied to properties such as the electron localization function (ELF)^9^ and the Laplacian of the density, ∇^2^ρ,^10^ which elucidate the role and locations of lone pairs and core and valence regions in chemical reactions. The Laplacian can also be used to delimit regions of strong and weak correlation^11^ and is crucial to the construction of kinetic energy functionals.^12−14^ The relationship between topology and the description of the overall system as a set of open quantum sub-systems, as initially demonstrated by Bader,^15^ has also been generalized.^16^

The use of topology to derive chemically intuitive quantities from first-principles calculations is an important part of strengthening the link between quantum mechanics and chemistry. However, it is also important to be able to move in the other direction—to be able to incorporate chemical ideas into first-principles calculations. Ideally, one would be able to set up a feedback loop whereby chemically intuitive quantities can be calculated from first-principles and fed back into the calculation to improve the results. This work investigates one route to achieve this for density functional theory (DFT) calculations by providing a method to calculate topological properties of functions defined on a real-space integration grid. This is achieved by the construction of a neighborhood graph over the DFT grid points and it is demonstrated that intrinsic properties of the graph, such as maximal spanning trees and strongly connected subgraphs, correspond to chemically relevant properties. Having such topological information available on a per-grid point basis allows for its direct incorporation into DFT calculations.

## 2 Topological Analysis on Arbitrary Grids

### 2.1 Terminology

A brief primer on notation and relevant mathematical concepts is provided in Appendix A. It is important to clarify that in what follows, and in the field of QCT more broadly, the term “topology” is used in a looser sense (with some exceptions—see ref (17)) than in the branch of mathematics bearing the same name. We use the term in its broader sense as pertaining to properties of a geometric object (in our case, a quantum-mechanical function) that are preserved under continuous deformations (in our case, small deformations of a molecule). In this work, the topological properties of interest will be properties of the *topography* of the quantum-mechanical function of interest. For example, maxima, minima, and saddle points are *topographical* features, but their existence and connectivity are *topological* properties. These topological properties are insensitive to the level of theory used to describe a molecule (e.g., Hartree–Fock or DFT). However, in contrast to stricter definitions of conservation in mathematics, topological properties in QCT are typically *not* conserved through chemical processes, such as bond breaking or formation—a fact which underpins their usefulness in identifying and classifying such phenomena.

### 2.2 Grids

In numerical studies, it is common to represent a function  by its values defined on a *gridG* of points in 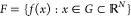
1

If the grid is constructed in a suitable fashion, it is possible to preserve information about the function in the neighborhood of a particular point. For example, if *G* is a uniform grid with spacing *s*
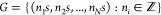
2then, we can define the neighbors of a particular grid point straightforwardly as
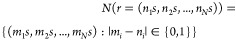
3

We can even go on to approximate the derivatives of *f* using, for example, finite differences
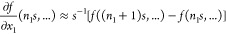
4assuming the spacing *s* is small enough to resolve variations in *f* accurately.

Despite the simplicity of a uniform grid, it is common to generate *G* in a less trivial fashion to reduce the storage requirements and the computational cost of operations on *F*. For example, in order to make routine DFT calculations feasible, typical grids used to perform real space integration become less dense further from atomic nuclei, where the electron density is lower and quantum-mechanical functions vary more slowly.^18^ Topological analysis on such non-uniform grids has been carried out previously in the context of Bader’s quantum theory of atoms in molecules (QTAIM^15^).^19−21^ However, such methods rely on the ability to freely evaluate *f* and its gradient ∇*f*. In the present work, a method to perform topological analysis without supplementing the function evaluations given in eq 1 is developed. This method is therefore a strict post-processing of *F* and can be easily applied to an arbitrary function (or set of data points with the form of eq 1). This also permits its packaging as a generally applicable software tool.^1^

### 2.3 Graphs over Grids

Determining the neighbors of a given grid point, as was done in eq 3 for a uniform grid, is a necessary prerequisite to perform a topographical analysis. Even simple topographical objects, such as local maxima and minima, are defined with reference to the behavior of the function when moving to “nearby” points. It is possible to encode the necessary information about the neighbors of a given grid point in the edges of a *neighborhood graphN* with nodes given by the points in *G*, and edges connecting each node *x* ∈ *G* to a set of neighbors *N*(*x*) ⊂ *G*/*x*. In this section, the construction of such graphs is investigated.

#### 2.3.1 Choice of Graph Construction

There are many ways to construct a neighborhood graph *N* for an arbitrary set of points *G* (a few are shown in Figure 2). In practice, *G* will be limited to a finite region of  and we will not be primarily concerned with the boundary of points forming the convex hull *H*(*G*), but only the bulk *B*(*G*) = *G*/*H*(*G*). The goal when choosing a construction is to most closely preserve the topography of *f* (and topology thereof) when moving from its representation in  to its representation on *G*. This leads to enforcing the following requirements for *N*

1 Connected: *N* should be connected (any node can be reached from any other node via a path along edges).
2 Undirected: *y* ∈ *N*(*x*) ⇒ *x* ∈ *N*(*y*).
3 Basis-preserving: Given *x* ∈ *B*(*G*), the vectors {*y* – *x*: *y* ∈ *N*(*x*)} must form a basis of .
4 Move-preserving: Given a point *x* ∈ *B*(*G*) and an arbitrary direction , a move can be made to a neighbor *y* ∈ *N*(*x*) so that the projection of the move onto δ is positive. In short, .

**Figure 1:**
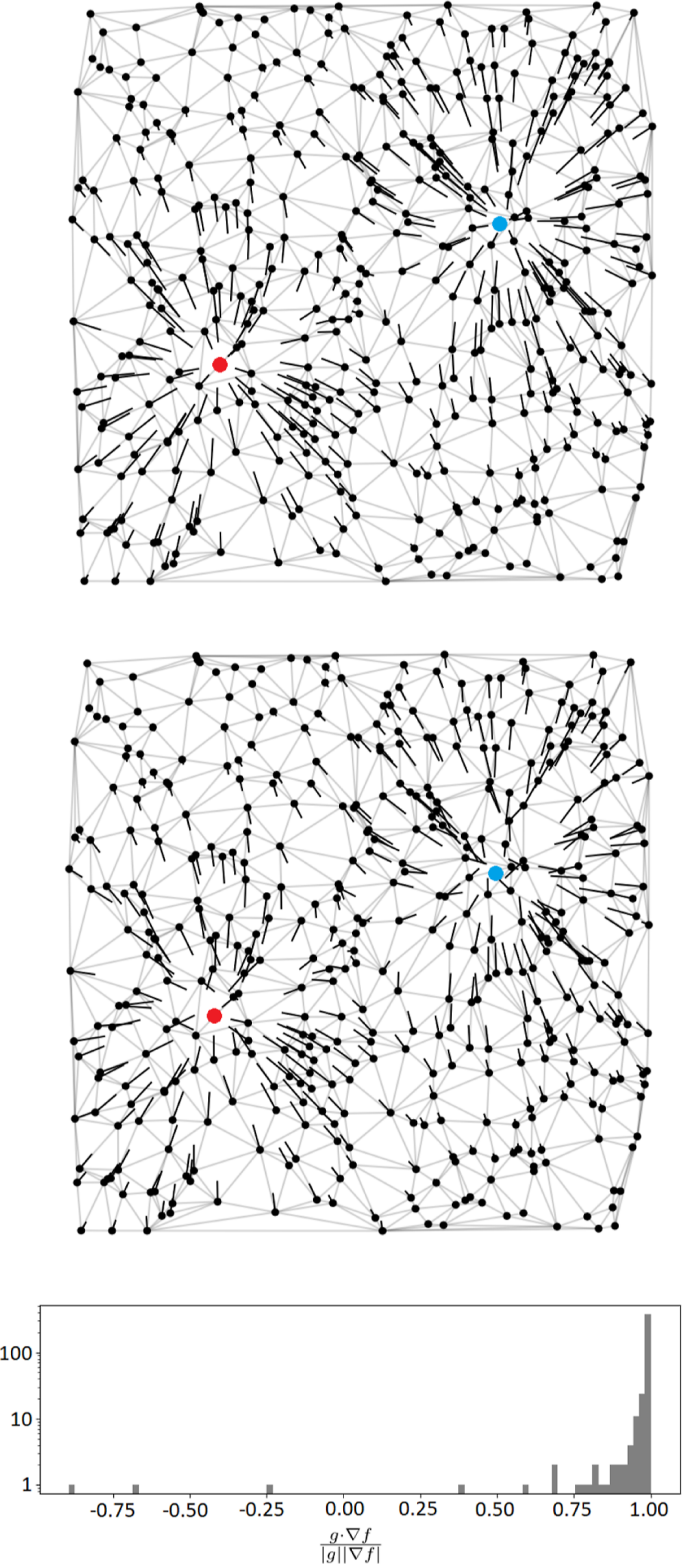
Analytic (∇*f*, top) and numerical (*g*, middle—calculated via eq 6) gradients for *f*(*x*) = exp(−|*x* – *a*|^2^) + exp(−|*x* – *b*|^2^) (*a* = red dot, *b* = blue dot) with neighbors given by a Delaunay triangulation (DT) (light gray graph) of a set of random points (black dots). A histogram (bottom, log scale) of normalized dot products between analytic and numerical gradients.

Condition 3 ensures the existence of an approximate gradient *g*(*x*) ≈ ∇*f*(*x*) on the graph via finite differences by minimizing the residual norm *∑*_*y*∈*N*(*x*)_|ϵ_*y*_|^2^ of a first-order Taylor expansion (see Figure 1 for an example)
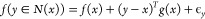
5leading to
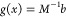
6where
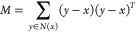
7
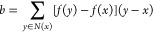
8Which would not have a unique solution (*M* would be singular) if {*y* – *x*: *y* ∈ *N*(*x*)} did not form a basis. Condition 3 is necessary, but not sufficient, for condition 4, which ensures that the gradient can be *followed* as well as approximated on the graph.

**Figure 2:**
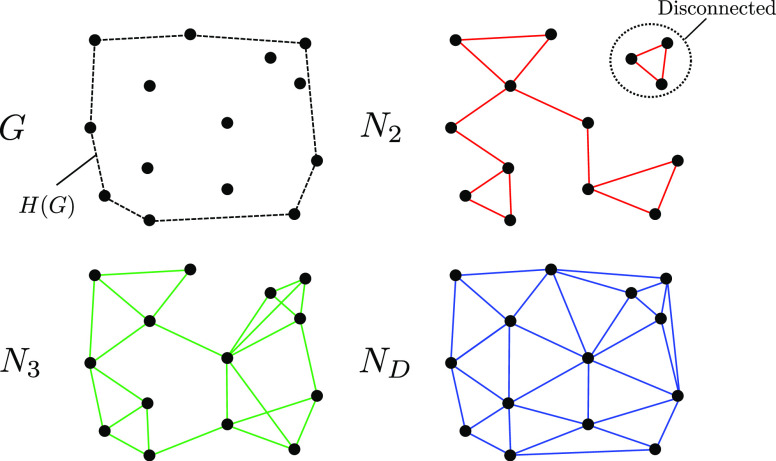
Three possible neighborhood graphs for the grid *G*. Graph *N*_2_ (red) is generated by connecting each grid point to its two nearest neighbors (note that the requirement of an undirected graph leads to more than two neighbors for some points). Graph *N*_3_ (green) is generated by connecting each grid point to its three nearest neighbors. These nearest neighbor graphs can lead to disconnected regions (as circled for *N*_2_) and nodes in the bulk that are not move-preserving (marked with black crosses). Graph *N*_D_ (blue) is a DT and exhibits no such issues.

**Figure 3:**
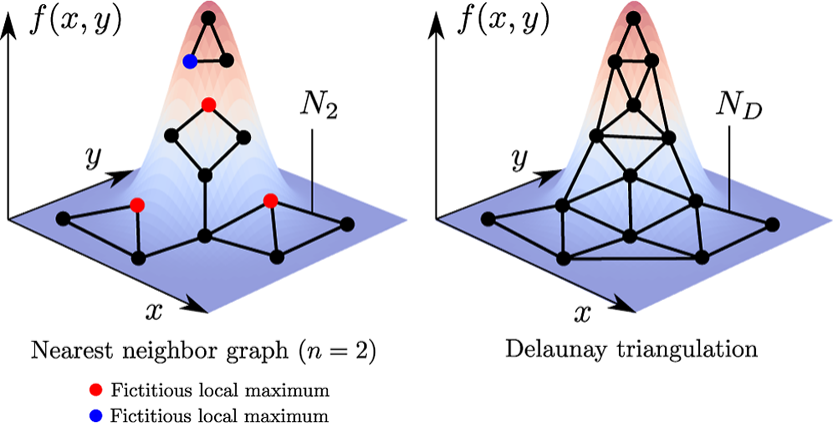
Function *f*(*x*, *y*) with a single maximum, represented on 2D graphs *N*_2_ (left) and *N*_D_ (right), constructed in the same way as those in Figure 2. The graph *N*_2_ introduces fictitious local maxima and minima (red and blue circles, respectively). *N*_D_ recovers the point closest to the true global maximum of *f* as the only local maximum.

These conditions serve to narrow down the choice of graph construction. For example, given the goal of defining a neighborhood, it might be tempting to use the set of *n* nearest neighbors of each point *N*^(*n*)^ (*x*) to define the *n*-nearest-neighbor graph
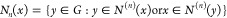
9where the reverse condition *x* ∈ *N*^(*n*)^ (*y*) has been included to ensure that the graph is undirected. Examples of nearest neighbor graphs *N*_2_ and *N*_3_ are shown in Figure 2 for a 2D grid, where we can see they suffer from several shortcomings. In particular, they are not necessarily connected or move-preserving which leads to the introduction of fictitious local maxima and local minima as can be seen in Figure 3.

#### 2.3.2 Delaunay Triangulation

A sensible choice of graph to overcome the issues with nearest-neighbor graphs is a *triangulation*. A triangulation of a grid *G* is a set of *simplices* (*N*-dimensional analogues of triangles) that tile the convex hull *H*(*G*) (see, e.g., *N*_D_ in Figure 2). Any triangulation immediately satisfies the requirements given in Section 2.3.1 and possesses high-quality numerical gradients, even for the pathological case of a random grid, as can be seen in Figure 1.

**Figure 4:**
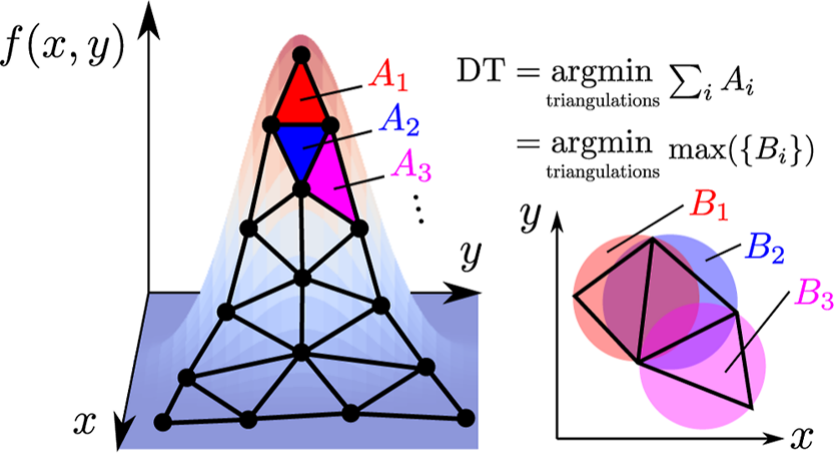
Illustration of the minimum-area and min-max-bounding-ball conditions satisfied by, and only by, a DT.

The specific case of a DT satisfies many additional desirable criteria,^22^ several of which also serve as independent definitions of the DT.^23^ Of particular relevance here, for a grid of points *G* and function evaluations *F*, the DT minimizes the area (volume for *d* > 2) of the polyhedral surface representing *F*—an illustration of this condition is shown in Figure 4 (left). The DT also minimizes the size of the largest open ball  which bounds a simplex^24^ and thus avoids large simplices corresponding to large neighborhoods—this is also shown in Figure 4 (lower right).

The choice of DT is related to the nearest-neighbor interpolation of the function
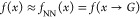
10where *x* → *G* is the nearest neighbor of *x* in *G*
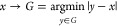
11

Given a grid point *y* ∈ *G*, the region , where *f*_NN_(*x*) = *f*(*y*) is known as the *Voronoi cell* of *y*. The neighborhood graph obtained via a DT is equivalent to connecting points with corresponding Voronoi cells that are adjacent in .^25^ Such connectivity of Voronoi cells of grid points is also known to be useful when approximating the flux of gradient paths between cells.^26^ We employ the QHull implementation of DT^27^ using the python interface provided by SciPy.^28^ Constructing the DT in *N*-dimensions is equivalent to constructing the convex hull of the points lifted into an *N* + 1 dimensional paraboloid—QHull constructs the DT by constructing this convex hull using the QuickHull algorithm.^27^

### 2.4 Maxima Families and Basins of Attraction

Along with a suitable definition for neighborhoods, it is important to be able to identify regions of interest in *G*. In particular, given a function , it is essential to be able to identify connected subsets of  for which *f* is locally maximal. These include not only pointlike maxima of *f* (e.g., the point *x* = 0 for *f* = −|*x*|) but also spatially extended maxima (e.g., the shell at |*x*| = 1 of *f*(*x*) = −(|*x*| – 1)^2^). Such a subset (and its analogue on *G*) will be referred to as a *maxima familyM* and the set of maxima families of *f* as . Then, for a given family , *f*(*x*) ≥ *f*(*x* + δ) ∀ *x* ∈ *M* for any infinitesimal perturbation . The concept of maxima families also permits the definition of *basins of attraction* of *f*. Given a starting point , we can define a *point* of attraction *A*(*x*) via repeated application of a steepest-ascent step
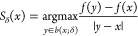
12as
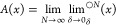
13where the open circle ○ in *S*_δ_^○*N*^ denotes *N* nested applications of *S*_δ_ to *x* (not taking the *N*^th^ power). A basin of attraction of *f* is then the region of  for which all steepest-ascent paths lead to a particular maxima family
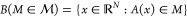
14

The concept of a steepest ascent path generalizes straightforwardly to a graph and so one might also expect basins of attraction to generalize straightforwardly. However, in general, the maxima of *f* will not lie exactly on the grid *G*. This means that the set of points on the graph that are best suited to represent a particular maxima family will not all have exactly the same function values and maxima families can only be approximately defined. In the present work, the definition is based upon an expansion around the *local maxima* of the graph *M*_L_(*G*) = {*x* ∈ *G*: *f*(*y*) < *f*(*x*) ∀ *y* ∈ *N*(*x*)} which are typically the closest points on *G* to maxima families of *f*. In order to construct the basins of attraction, two objects must be constructed; *A*: *G* → *M*_L_(*G*) which maps a point to the local maximum whose basin of attraction it resides in [in analogy to the point of attraction ] and the families of local maxima  [in analogy to the maxima families  on ]. The basins of attraction for the maxima families are then
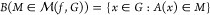
15in analogy with eq 14.

**Figure 5:**
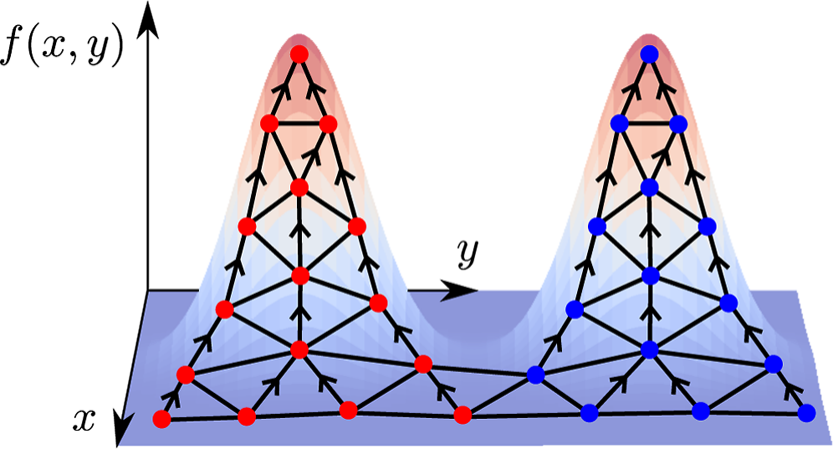
Schematic showing how steepest ascent paths (arrows) on a graph allow us to reconstruct the two regions of attraction (the set of red and blue dots, respectively) for two separate maxima of the same function.

The algorithm to determine *A* is based on that of Henkelman et al.,^29^ but applied to a graph rather than to a uniform grid. A schematic is shown in Figure 5 and the steps are detailed below

1 Initialize: Let *D*(*A*) be the domain of *A*: *G* → *M*_L_(*G*) (i.e., the set of points assigned to a local maximum). Initially, *D*(*A*) = ϕ.
2 Check termination: If the set of unassigned points *G*/*D*(*A*) is empty, then *A*: *G* → *M*_L_(*G*) is complete on *G* (all points have been assigned) and the algorithm terminates.
3 New path: Identify an unassigned point *x* ∈ *G*/*D*(*A*) and start a steepest ascent path *P* = {*x*}.
4 Reached maxima: If *x* ∈ *M*_L_(*G*), then let *A*(*p*) = *x* ∀ *p* ∈ *P* and return to step 2
5 Steepest step: Identify *y* ∈ *N*(*x*) that maximizes [*f*(*y*) – *f*(*x*)]/|*y* – *x*| and add it to *P*.
6 Shortcut: If *y* is assigned [*y* ∈ *D*(*A*)], then let *A*(*p*) = *A*(*y*) ∀ *p* ∈ *P* and return to step 2.
7 Iterate: Let *x* = *y* and return to step 4.

As noted in ref (30), step 6 of this algorithm allows rediscovery of previous steepest ascent paths and significantly improves runtime performance.

Once we have constructed the map *A*: *G* → *M*_L_(*G*) associating points to local maxima, we turn our attention to the algorithm to cluster local maxima into families . This clustering is based upon a measure of deviation  that increases as *y* moves away from the maxima family containing the local maximum *x*. In the present work, the following measure is used
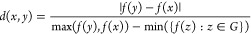
16

**Figure 6:**
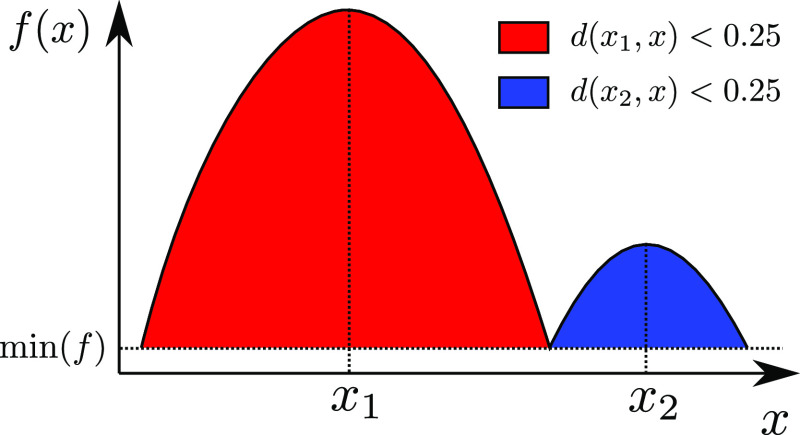
Regions with a deviation *d*(*x*_*i*_, *x*) of less than *t* = 0.25 for two maxima *x*_1_ and *x*_2_ of a function *f*(*x*). Note that the region for the smaller maxima is smaller, thanks to the scale-independence of the deviation measure (eq 16).

**Figure 7:**
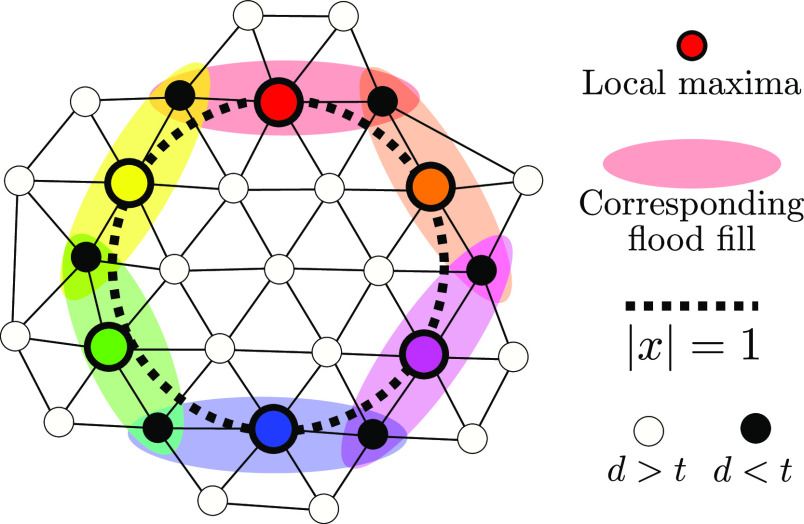
Schematic showing how the algorithm in Section 2.4 identifies the circular maxima family of the function *f*(*x*) = −(|*x*| – 1)^2^. Nodes that are within (beyond) *t* of a local maximum according to the measure of deviation are shown as black (white) circles. Note that all of the flood fills overlap, leading to all of the local maxima being considered as part of the same maxima family.

This is essentially the fractional change in the function value due to moving from *x* to *y*, and therefore, *d*(*x*, *y*) ∈ [0, 1] independently of the scale of the function. Once *d*(*x*, *y*) has been defined, a tolerance *t* can be chosen such that *d*(*x*, *y*) < *t* defines a stationary region around each maxima (see Figure 6) and apply the following algorithm to cluster local maxima into families. The algorithm begins by constructing a flood fill around each local maxima according to the tolerance and ends by merging overlapping flood fills into connected families (see Figure 7)

1 Initialize: Let *i* = 0 and *F*_0_ = ϕ be an empty flood fill.
2 New maxima: Identify a local maximum that is not yet in a flood fill *x* ∈ *M*_L_(*G*)/∪_*j*_*F*_*j*_ and let *F*_*i*_ = {*x*}. If no such maxima exist, go to step 5.
3 Identify shell: Identify the shell *S* of neighbors surrounding *F*_*i*_ as . Identify the points in the shell that are still within tolerance of the initial maximum *T* = {*y* ∈ *S*: *d*(*x*, *y*) < *t*}. If *T* is empty, then *F*_*i*_ is complete; set *i* = *i* + 1 and return to step 2.
4 Expand: Expand *F*_*i*_ to include points in *T*: *F*_*i*_ → *F*_*i*_ ∪ *T*. Return to step 3.
5 Merge floods: If any two flood fills overlap (∃ *i* ≠ *j*: *F*_*i*_ ∩ *F*_*j*_ ≠ ϕ), then merge into a single flood fill: *F*_min(*i*,*j*)_ → *F*_*i*_ ∪ *F*_*j*_, *F*_max(*i*,*j*)_ → ϕ. Repeat this process until no flood fills overlap.
6 Assign families: Group maxima into families according to the merged flood to which they belong .

### 2.5 Basins of Attraction: Example Applications

#### 2.5.1 Calculation Parameters

Unless stated otherwise, example applications presented in the rest of this work were carried out using quantities from a Hartree–Fock calculation with a cc-pVDZ basis set. For topology analysis, the quantity of interest is then evaluated on a DFT grid generated using an Lindh–Malmqvist–Gagliardi (LMG) radial grid^31^ (with a threshold of 10^–10^), a Lebedev angular grid^32^ (with degree between 15 and 25 depending on the radius), and by pruning points with a weight of less than 10^–12^. This results in a relatively coarse DFT grid (∼10^4^ points per atom), with the hope of replicating the worst-case scenario that would be encountered in real-world applications. Hartree–Fock was used rather than DFT so that the dependence of the topology analysis on the grid could be investigated independently of the quality of Fock-matrix integration (for which the DFT grid is used).

#### 2.5.2 Bader Regions

**Figure 8:**
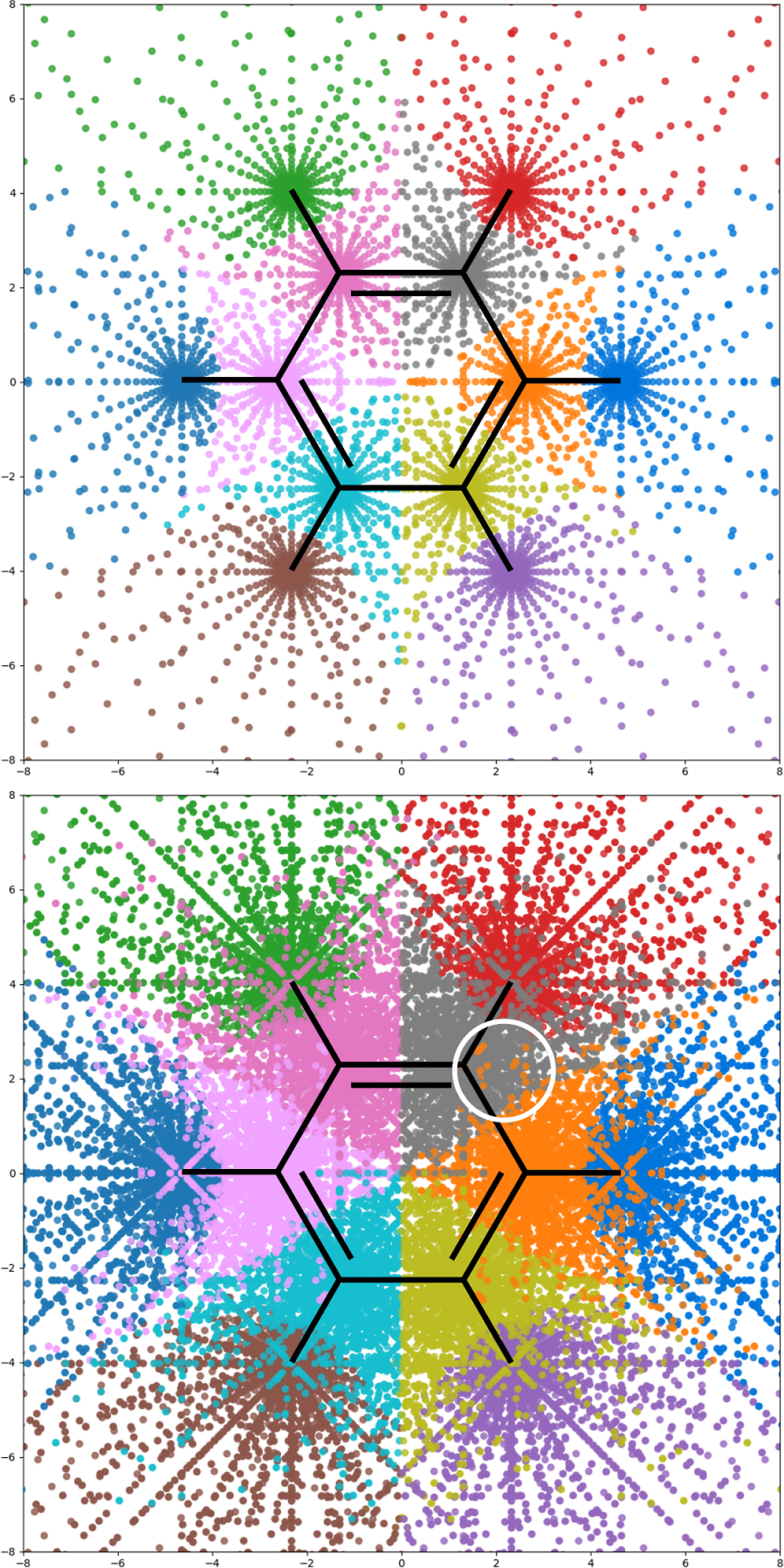
DFT grid points for benzene colored according to basins of attraction of the electron density, evaluated by the algorithm given in Section 2.4. The top figure shows only points within 0.01 bohr of *z* = 0 and demonstrates the proper sixfold symmetry of the regions. The bottom figure shows all points, including some which have been misassigned due to the sparsity grid points far from the nuclei (e.g., the orange points highlighted with a white circle at *z* > 5 bohr, where +ve *z* is out of the page).

An object of central importance in quantum chemistry is the electron density . Bader demonstrated a correspondence between the basins of attraction of the electron charge density and atoms in molecules.^15^ Specifically, each basin of attraction contains exactly one atom in a molecular system, allowing one to uniquely assign the electronic charge present on each atom as the integral of the charge density over its basin of attraction. This leads to the *Bader charges*, here defined in  as
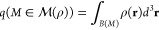
17and on *G* as
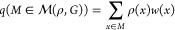
18where *w*(*x*) are grid integration weights (typically generated along with the grid itself,^18^ but which could be taken as the volume of the Voronoi cell of *x*). The basins of attraction for the electron density of a benzene molecule are shown in Figure 8. Near to the *z* = 0 plane (Figure 8, top), the basins of attraction are delimited into six wedges, each containing a single H basin and a single C basin according to the sixfold rotational symmetry of benzene. However, further from the nuclei some points are assigned to basins of attraction outside of their wedge (Figure 8, bottom, white circle). For the coarse grids specified in Section 2.5.1, this misassignment affects approximately 1% of the points. However, as these points are in regions of low electron density, the error in Bader charges resulting from this misassignment is of the order of 1/1000th of an electron. The convergence of Bader charges as a function of grid size is investigated in detail in Section 2.8.1.

#### 2.5.3 Electron Shells from ∇^2^ρ

**Figure 9:**
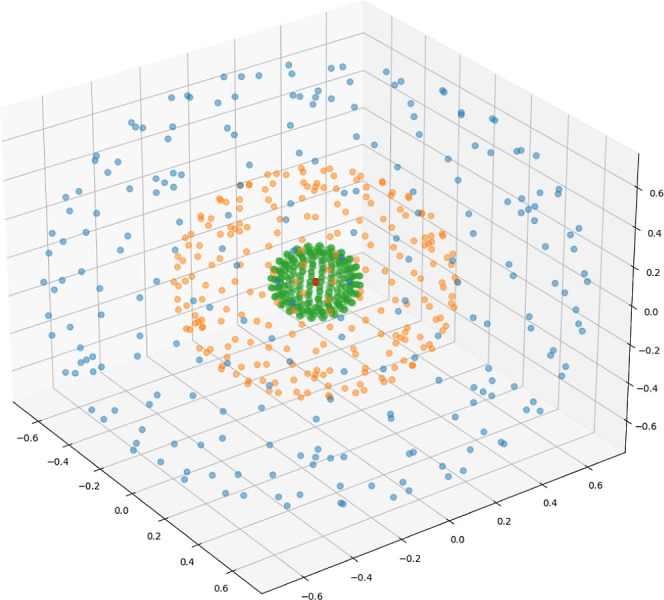
Atomic shells of a Ne atom, visualized by plotting the distinct maxima families of |∇^2^ρ| on a DFT grid, identified by the algorithm given in Section 2.4. The axes are in bohr.

Bader charge analysis as carried out in Section 2.5.2 is insensitive to the treatment of maxima families. This is because, for molecular systems, the electron density ρ has no extended maxima, only distinct point-like maxima near to each nucleus. However, this is not true for the Laplacian of the electron density ∇^2^ρ. Indeed, the electronic shell structure of atoms leads to ∇^2^ρ exhibiting alternating regions of charge concentration (∇^2^ρ < 0) and charge depletion (∇^2^ρ > 0) as one moves radially away from the nucleus.^10^ This naturally leads to spatially extended maximal shells of ∇^2^ρ and derived quantities, as can be seen for a Neon atom in Figure 9. The changes in the shell structure of the Laplacian upon bond formation will be discussed in Section 2.7.2.

#### 2.5.4 Isosurfaces

**Figure 10:**
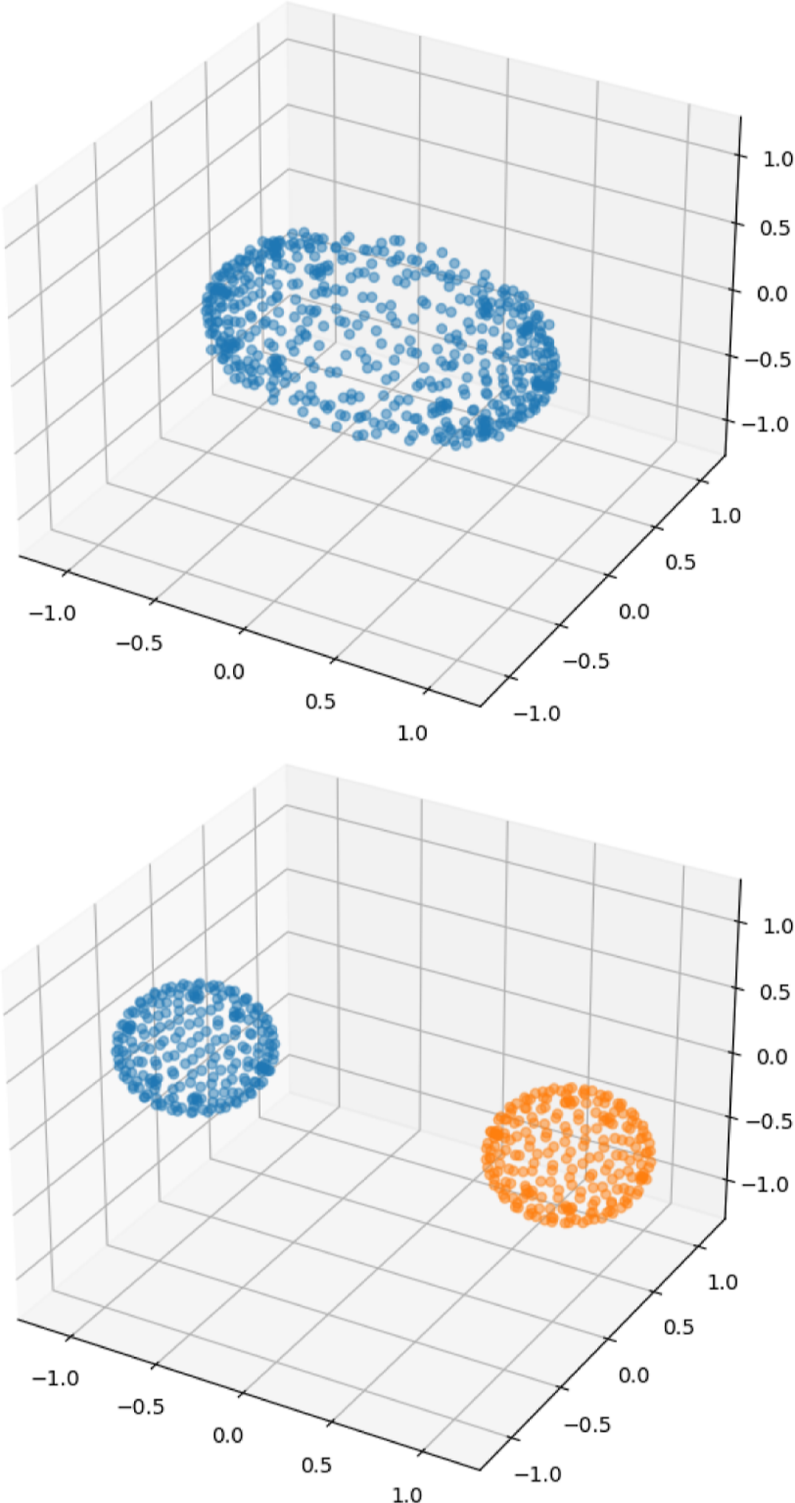
Plots of the density isosurface(s) ρ(*x*) = ρ_iso_ = max(ρ)/2 for the H_2_ molecule under a magnetic field of 1 *B*_0_ perpendicular to the bond, obtained as the maxima families of the auxiliary function ρ_I_(*r*) = exp(−|ρ(*r*) – ρ_iso_|). Top: covalent bonding in the singlet 1σ_g_^α^1σ_g_^β^. Bottom: Non-covalent bonding of the 1σ_g_^β^1σ_g_^β^ component of the triplet state (which, under this magnetic field, is the ground state^33^). The axes are in bohr.

Given a target function value *f*_iso_, an isosurface of *f* can be defined as . Due to the delocalized nature of electrons in molecules, isosurfaces are commonly used in molecular visualization. The ability to identify families of maxima allows the topological analysis of such isosurfaces by defining an auxiliary function *f*_I_(*x*) = exp(−|*f*(*x*) – *f*_iso_|) which will be maximal, where *f*(*x*) = *f*_iso_. The maxima family (or families) where *f*_I_(*x*) ≈ 0 then serve as a suitable definition of isosurfaces. An example of this can be seen in Figure 10, where non-covalent bonding in H_2_ under a strong magnetic field (as explored in ref (33)) can be identified as the separation of the half-maximum-value isosurface of the electron density (where ρ(*x*) = max(ρ)/2) into two distinct maxima families.

### 2.6 Critical Paths

**Figure 11:**
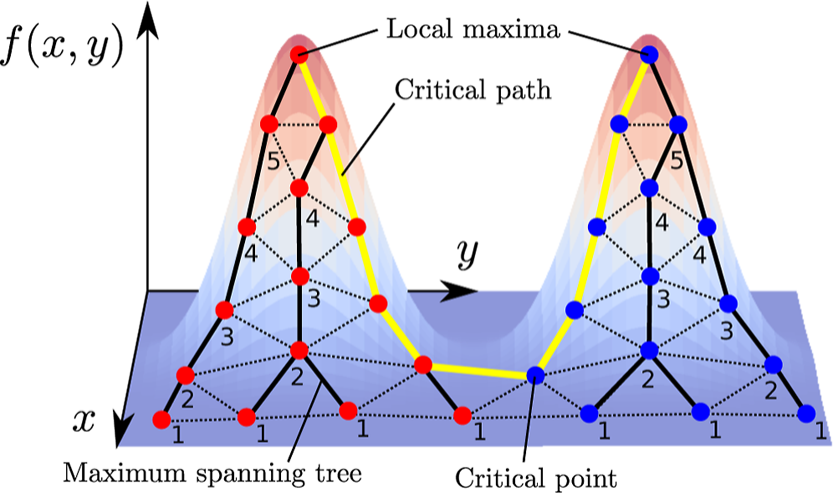
Schematic demonstrating how critical paths can be identified by pruning the maximum spanning tree of a graph according to the algorithm presented in Section 2.6. Nodes that are pruned are labeled by the algorithm iteration number at which they are pruned.

A critical path is defined as a path linking two local maxima on *N* that maximizes the minimum value of *f* encountered (the *critical value* of that path). The equivalent of this path in  necessarily passes through a first-order saddle point of *f* known as a *critical point* and, in analogy, the point of minimum *f* on a critical path in *N* is labeled as a critical point (see Figure 11). Given a neighborhood graph *N*, edge weights are assigned as the average of the function values at the endpoints of the edge. It is then possible to find critical paths rapidly by noting that they are paths on the *maximum spanning tree* (MST) of *N* (see Appendix A), which is denoted as *M*(*N*) (the critical-path problem essentially becomes the *widest path* problem from graph theory). In fact, the critical path between two local maxima on *N* is the *only* path linking the maxima on *M*(*N*), thanks to the fact that *M*(*N*) is acyclic. The union of all critical paths is called the *critical tree*, which can be found rapidly and in its entirety by repeatedly pruning the maximum spanning tree according to the following algorithm (shown in Figure 11)

1 Maximum spanning tree: Let *M* be the maximum spanning tree of *N*, evaluated with edge weights given by the average of function values on the endpoints of each edge.
2 Identify leaf nodes: Let *C* be the set of leaf nodes of *M*(*N*) (nodes with only one neighbor) that are not local maxima. If there are no such nodes, terminate the algorithm.
3 Pruning: Remove the nodes *C* from *M*. Return to step 2.

One can avoid searching the entire tree for leaf nodes at every iteration of step 2 by expanding from the previous set of pruned leaf nodes.

#### 2.6.1 Recovering Cyclic Graphs (Gap-Filling)

**Figure 12:**
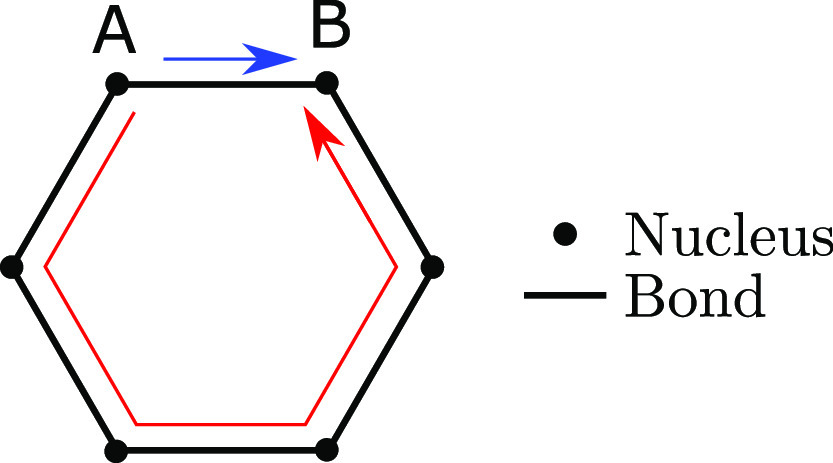
Two paths (red and blue arrows), with similar critical values, connecting nuclei A and B around the bonding network of a benzene molecule.

The critical tree is inherently acyclic (as it is a subgraph of the maximum spanning tree)—a direct consequence of the definition of a critical path. However, it is possible that there are multiple paths with very similar critical values between a given pair of local maxima. For example, the electronic charge density of a benzene molecule exhibits local maxima at the nuclei which can be linked together by traversing the aromatic ring either clockwise, or anticlockwise (see Figure 12). Both of these routes have very similar critical values, but only one (that which has the slightly larger critical value within a finite precision computation) will be included in the critical tree. For the benzene system this means that whichever bond happens to have the lowest electron density will be excluded from the maximum spanning tree and hence also from the critical tree. However, such bonds can be re-introduced by considering neighboring basins of attraction using the following gap-filling algorithm (this produces a *critical network* according to the definition of Bader^8^)

1 Initialize: Let *C* be the critical tree (as determined via the above algorithm).
2 Iterate: Iterate over pairs of maxima *x*, *y* ∈ *M*_L_(*G*).
3 Check already linked: If the path between *x* and *y* on *C* passes through only two basins of attraction, then *x* and *y* are already critically linked in *C* and we can continue to the next iteration (go to step 2).
4 Identify basins: Let *B*_*x*_ (*B*_*y*_) be the basin of attraction containing the point *x* (*y*).
5 Check neighboring: If the basins *B*_*x*_ and *B*_*y*_ are not adjacent (i.e., , where *N*(*z*) are the neighbors of *z* in *G*), then *x* and *y* are not critically linked. Continue to the next iteration (go to step 2).
6 Construct subgraph: Construct the subgraph of *G* containing only nodes in the basins of attraction *B*_*x*_ and *B*_*y*_ as *G*_*xy*_ = *G* ∩ (*B*_*x*_ ∪ *B*_*y*_) and its boundary *B*(*G*_*xy*_) = {*z* ∈ *G*_*xy*_: ∃ *z*_2_ ∈ *N*(*z*) s.t *z*_2_ ∉ *G*_*xy*_}.
7 Construct MST: Construct the maximum spanning tree *M*_*xy*_ of *G*_*xy*_. Identify the path *P*_*xy*_ linking *x* and *y* on *M*_*xy*_.
8 Reject non-critical path: If, at any point, the path *P*_*xy*_ touches the boundary (i.e., *P*_*xy*_ ∩ *B*(*G*_*xy*_) ≠ ϕ), then *x* and *y* are not critically linked. Continue to the next iteration of the loop (step 2).
9 Fill gap: *P* is a critical path in *G*_*xy*_ linking *x* and *y* and the edges along *P* should be added to *C*. Continue to the next iteration of the loop (step 2).

**Figure 13:**
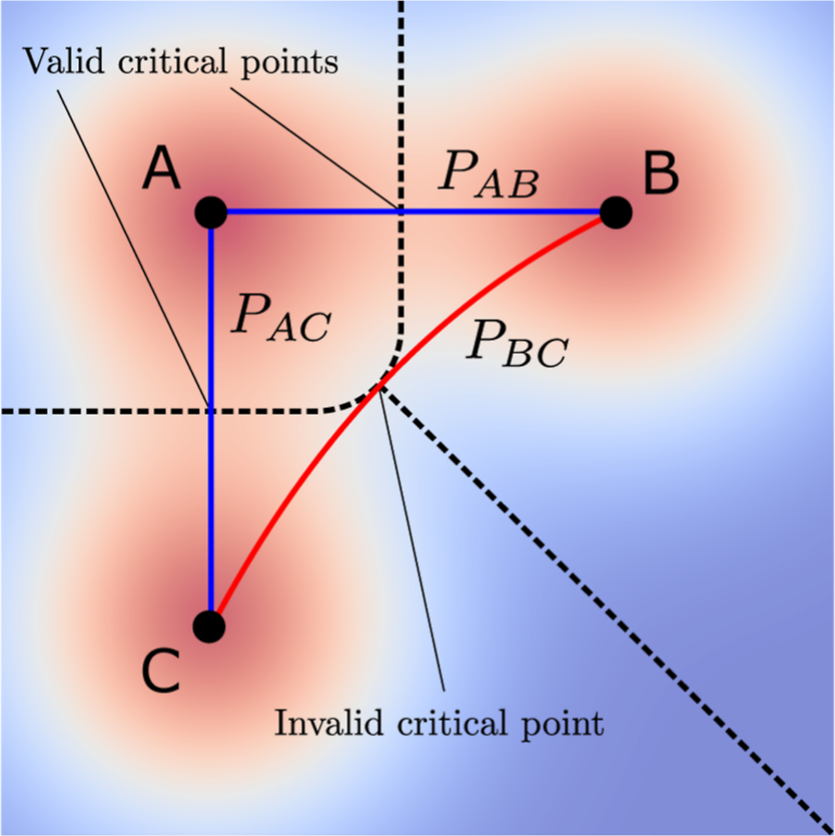
Schematic of critical (*P*_*AC*_ and *P*_*AB*_, blue) and non-critical (*P*_*BC*_, red) paths linking three maxima of a function on the plane (whose basins of attraction are separated by dashed lines). Note that the non-critical path between *B* and *C* touches the boundary of the union of their basins of attraction.

Step 8 identifies a non-critical path between neighboring regions by noting that the critical point is pushed right to the edge of the shared border of the regions (see path *P*_*BC*_ in Figure 13). In order for two regions to be critically linked, the critical point must instead constitute a saddle point in the bulk of the shared boundary (as is the case for paths *P*_*AC*_ and *P*_*AB*_ in Figure 13).

We could have generated *all* of our critical paths by following this gap-filling algorithm by starting instead with an empty graph *C*. However, starting with the critical tree avoids having to construct the subgraph *G*_*xy*_ for every pair *x*, *y* and is thus more efficient.

#### 2.6.2 Cleaving

By construction, the critical tree contains and connects all local maxima in the network. However, as we will see later, it is useful to be able to divide the critical tree into sub-trees within which *f*(*x*) varies only weakly. This process is called *cleaving* and it proceeds as follows

1 Initialize: Let *C* be the critical tree of *f*(*x*) on *G* and *P*(*x*, *y*) be the path between points *x* and *y* on *C*.
2 Get paths: Let *P* be the set of all critical paths in *C* (i.e., paths between local maxima that do not pass through other local maxima): .
3 Set function scales: For each path, set a function scale as the maximum endpoint value *f*_scale_(*P*(*x*, *y*)) = max(*f*(*x*), *f*(*y*)).
4 Calculate deviations: For each path *P*(*x*, *y*), calculate a deviation

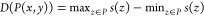
19where *s*(*z*) is the function value, scaled so the maximum endpoint value is 1
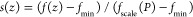
20and *f*_min_ = min{*f*(*a*): *a* ∈ *G*} is the global minimum function value.

5 Cluster paths: Cluster the paths into a *flat* set , where the function value changes by a small amount (according to some tolerance *t*_flat_) along the path. Consider the rest of the paths to be *non-flat*. In the present work, a kernel density estimate^34^ of the distribution of deviations  is used to inform the choice of cluster tolerance *t*_flat_.
6 Cleave non-flat paths: Remove the edges of each *non-flat* path from *C*.

In an alternative scheme, one might use the subgraphs of the cleaved critical tree to define the maxima families when identifying basins of attraction. However, the flood fill technique introduced in Section 2.4 is more robust in practice (as the flood fills are more densely connected over surface-like maxima than a tree).

### 2.7 Critical Paths: Example Applications

#### 2.7.1 Bond Paths

**Figure 14:**
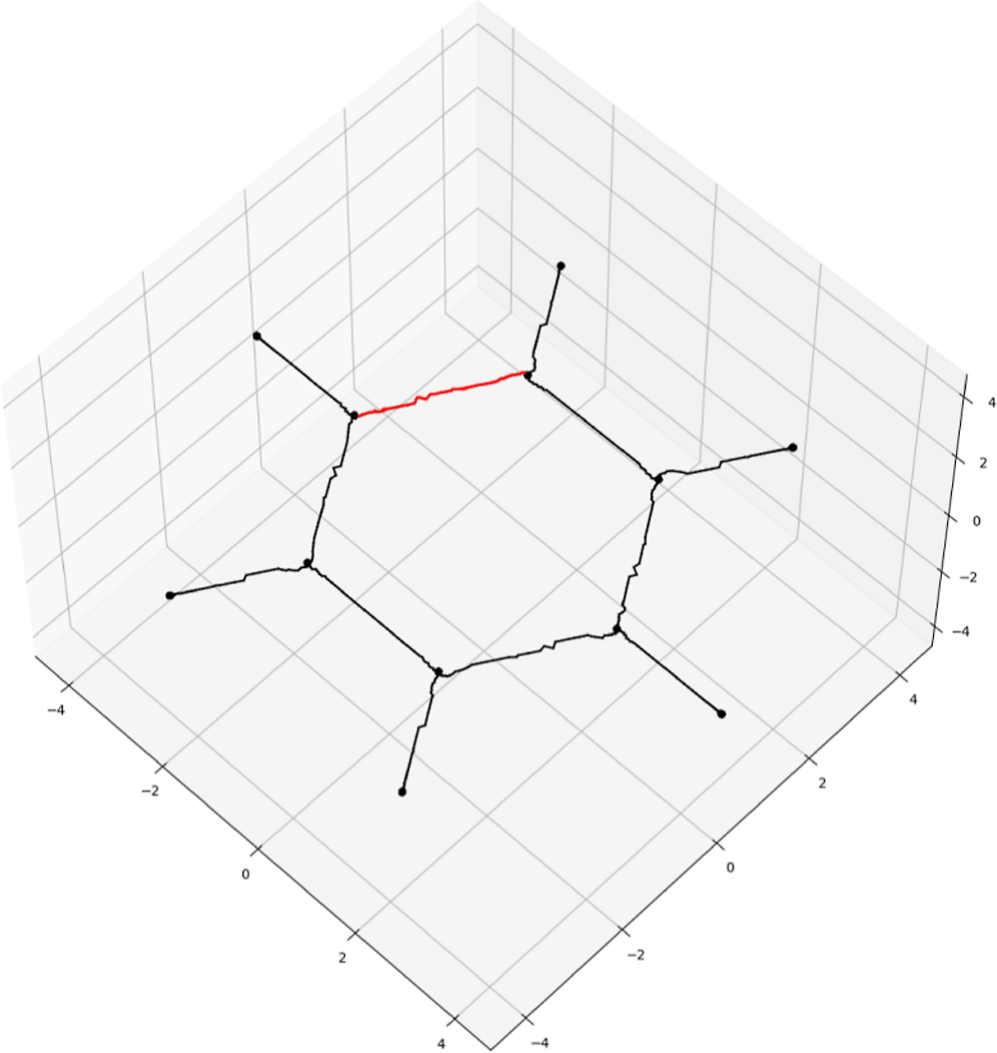
Critical network of the electron density of a benzene molecule, evaluated by post-processing the maximum spanning tree on a DT according to the algorithms given in Section 2.6. The bond that was filled in by the gap-filling algorithm is shown in red; the remaining black lines are the pruned maximum spanning tree. To improve the smoothness of the bonds, the DFT grid used for this plot contains around twice the number of grid points as the coarser grids used in the rest of this work. The axes are in bohr.

In Bader analysis, paths on the critical network are called *bond paths*, and provide a unique (although not necessarily optimal^2^) definition of molecular bonds.^8^ The bond paths for benzene, evaluated using the algorithm given in Section 2.6, are shown in Figure 14. All bonds are recovered (one of which via the gap filling algorithm given in Section 2.6), leading to the familiar hexagonal benzene bonding network. In Bader analysis, the critical points are known as *bond critical points*—the values of the electron density at these points are given in Table 1 alongside values calculated using existing methods that require ρ(**r**) at arbitrary **r**.

**Table 1:** Values of the Charge Density (in e/bohr^3^) for Each Bond Critical Point Identified in Benzene for Named Grid Sizes in QUEST^a^

grid	coarse	standard	fine	ultrafine	Multiwfn
points	102198	222314	420238	971062	82 million
C–H bonds	0.29263	0.29466	0.29448	0.29443	0.29440
	0.29263	0.29466	0.29448	0.29443	0.29443
	0.29263	0.29494	0.29461	0.29478	0.29472
	0.29263	0.29494	0.29461	0.29478	0.29472
	0.29443	0.29494	0.29461	0.29478	0.29476
	0.29443	0.29494	0.29461	0.29478	0.29477
std. dev.	0.00085	0.00013	0.00006	0.00016	0.00016
C–C bonds	0.31146	0.31598	0.31664	0.31673	0.31630
	0.31146	0.31598	0.31664	0.31673	0.31630
	0.31146	0.31598	0.31664	0.31673	0.31630
	0.31146	0.31598	0.31664	0.31673	0.31630
	0.31640	0.31656	0.31732	0.31693	0.31640
	0.31640	0.31656	0.31732	0.31693	0.31641
std. dev.	0.00233	0.00027	0.00032	0.00009	0.00005

a The values can be clearly seen to be split into groups of 4 and 2 as a result of the DFT grid breaking sixfold symmetry. The level of theory is as specified in Section 2.5.1 and results using QChem v5.0^35^ and the Multiwfn package v3.8^36^ are also given.

#### 2.7.2 Valence Charge Concentration and Depletion Graphs

**Figure 15:**
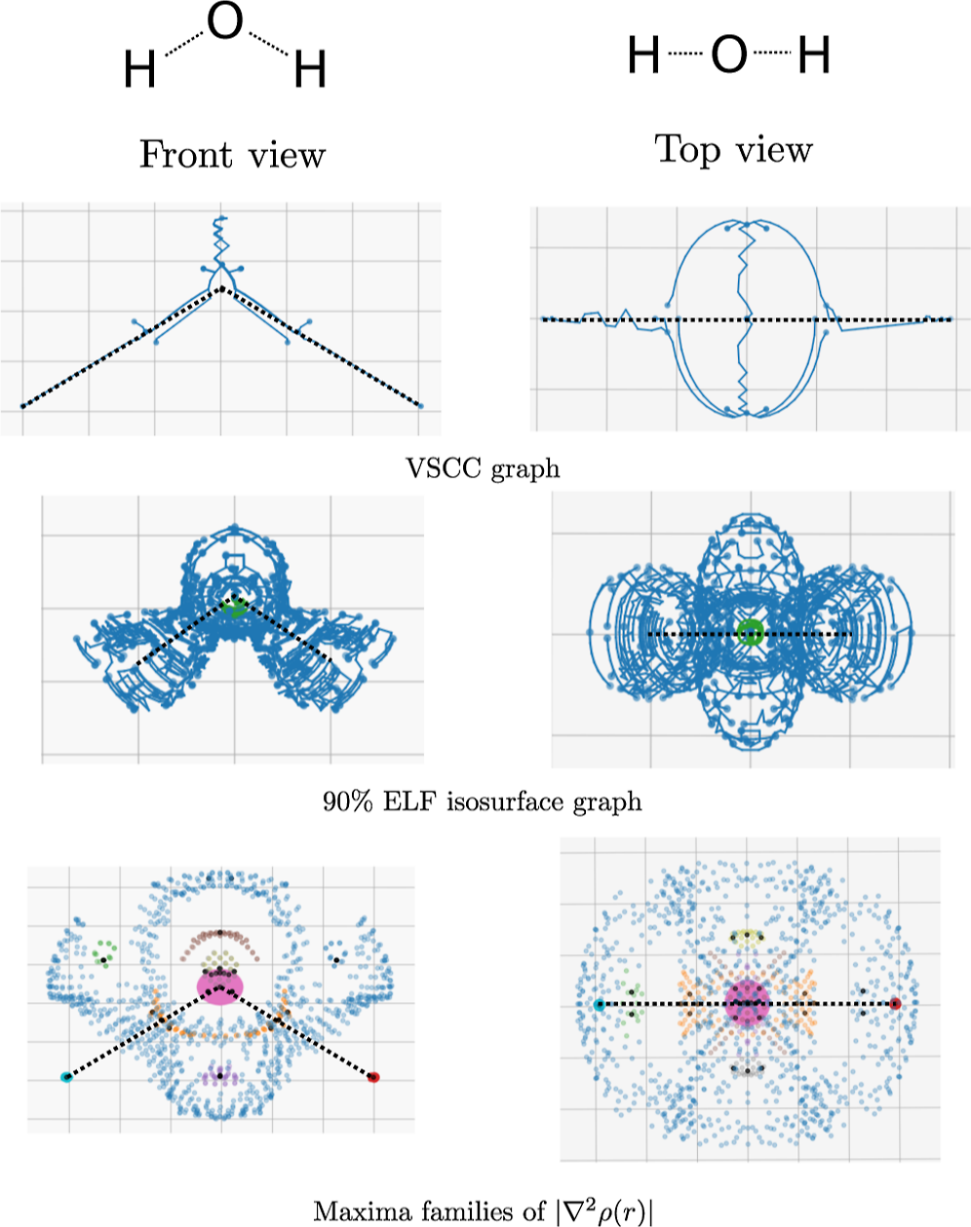
Topographical analysis for various properties of the water molecule. The molecular geometry is shown as a dotted line.

Charge concentration (∇^2^ρ < 0), or depletion (∇^2^ρ > 0), is most relevant to chemistry when it occurs in the valence region of an atom in a molecule. In particular, it has been noted that the maxima of valence charge concentration (depletion) correlate with the active regions for electrophilic (nucleophilic) attack.^37^ Critical networks spanning these maxima form the valence shell charge concentration/depletion (VSCC/D) graphs.^10^ Such graphs can be easily examined by constructing the critical network of −∇^2^ρ (charge concentration) or ∇^2^ρ (charge depletion). An example for the VSCC graph of a water molecule is shown in Figure 15 (top, cf. Figure 3 of ref (10)). This VSCC graph can clearly be seen to connect the lone pairs either side of the oxygen atom. This behavior is reflected in the critical network of the 90% ELF isosurface (Figure 15, middle), where the lone pairs can be very clearly seen as lobes aligned along the perpendicular direction to the bonds. Such charge concentration arises from distortions in the valence shell of the oxygen atom due to the hydrogen atoms, which can be seen by looking at the maxima families of |∇^2^ρ(**r**)| (Figure 15, bottom, valence shells are shown in blue, cf. the shells of Ne in Figure 9)—note that core shells (pink, e.g.) retain their spherical nature.

#### 2.7.3 Stagnation Graphs

**Figure 16:**
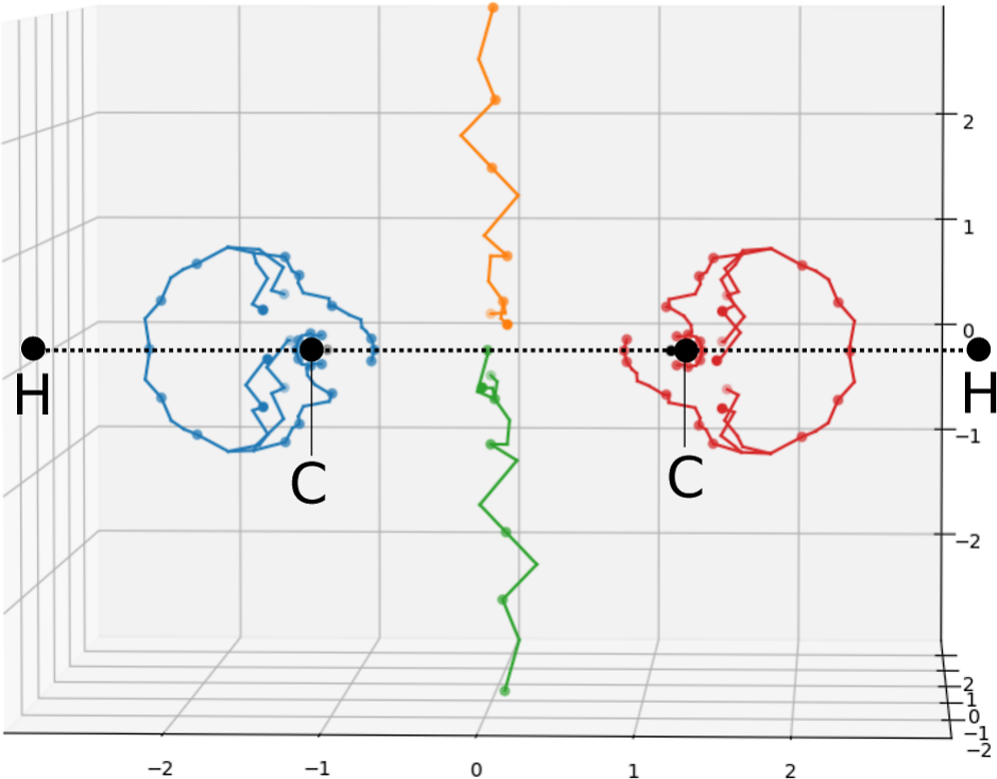
Stagnation graph of C_2_H_2_, visualized as the cleaved critical tree of −|**J**|. The axes are in bohr.

Applying a magnetic field to a molecule induces a current density vector field . The *stagnation graph* of **J** is the subset of  where |**J**(*x*)| = 0 and in general consists of isolated stagnation points and extended stagnation lines.^38^ These stagnation graphs form a compact representation of the topology of the vector field^39^ and have significance in ring-current models and NMR spectra.^40^ The stagnation graph can be obtained as the critical network of −|**J**|, as can be seen for a C_2_H_2_ molecule in Figure 16. This stagnation graph exhibits the same features as a more detailed analysis at significantly reduced cost.^41^ The graph is known to contain a stagnation line that bisects the molecule—this can be seen in Figure 16, but is quite ragged due to the decreasing density of DFT grid points as we move further from the nuclei. In combination with this, the single-valued (|**J**| = 0) and line-like (and therefore weakly connected) nature of stagnation graphs make for a challenging test case for topological analysis. In any case, the approximate stagnation points determined via the present analysis on DFT grids can be used as a starting point for the derivative-based optimization and refinement of the stagnation graph presented in ref (41). Utilizing the starting points from the algorithms in the present work can significantly reduce the cost of determining detailed stagnation graphs using the derivative-based approaches.

### 2.8 Performance

#### 2.8.1 Convergence

**Figure 17:**
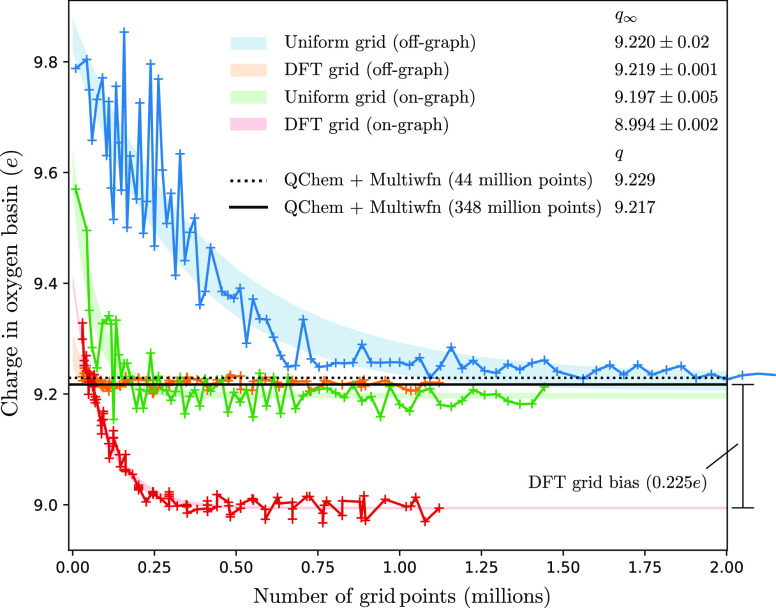
Convergence properties of the Bader charges of the oxygen basin in a water molecule for different grids and graph ascent methods. Data points are shown as crosses connected by solid lines and the region within one standard deviation of an exponential fit is shaded for each series. The infinite grid density limit is given for each series as *q*_∞_, along with the fitting error. Uniform grids are scaled by decreasing the grid spacing, DFT grids are scaled by reducing the LMG tolerance and increasing the Lebedev degree simultaneously. The charge density was calculated using HF with a primitive aug-cc-pVDZ basis in Cartesian representation. Results obtained at the same level of theory using QChem v5.0^35^ and the Multiwfn package v3.8^36^ are also shown—the same numbers are obtained if Psi4 v1.6.1^42^ is used in the place of QChem.

**Figure 18:**
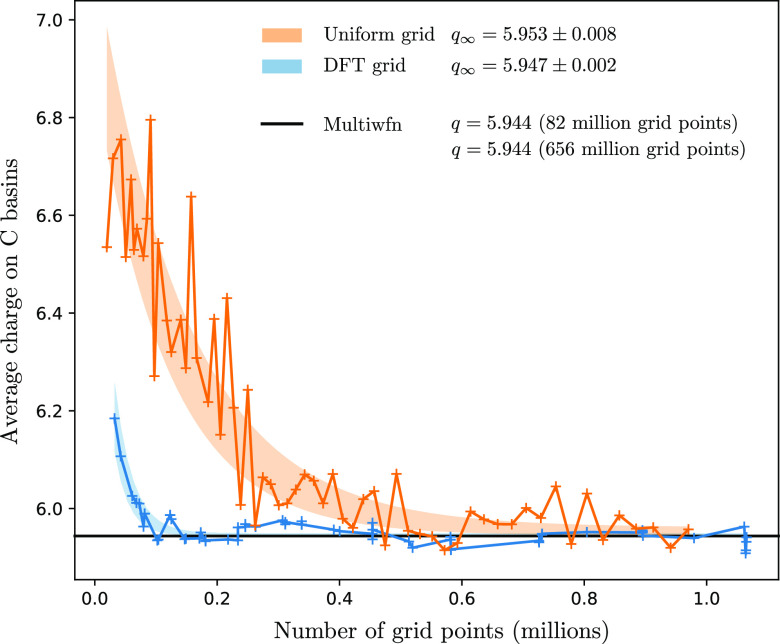
As Figure 17, but for the average of carbon basins in benzene. The charge density is calculated following the method outlined in Section 2.5.1. Only off-graph results are shown. In contrast to the water case, the difference in Multiwfn results for the “lunatic” and “extra-lunatic” grids is not resolvable on this scale.

**Figure 19:**
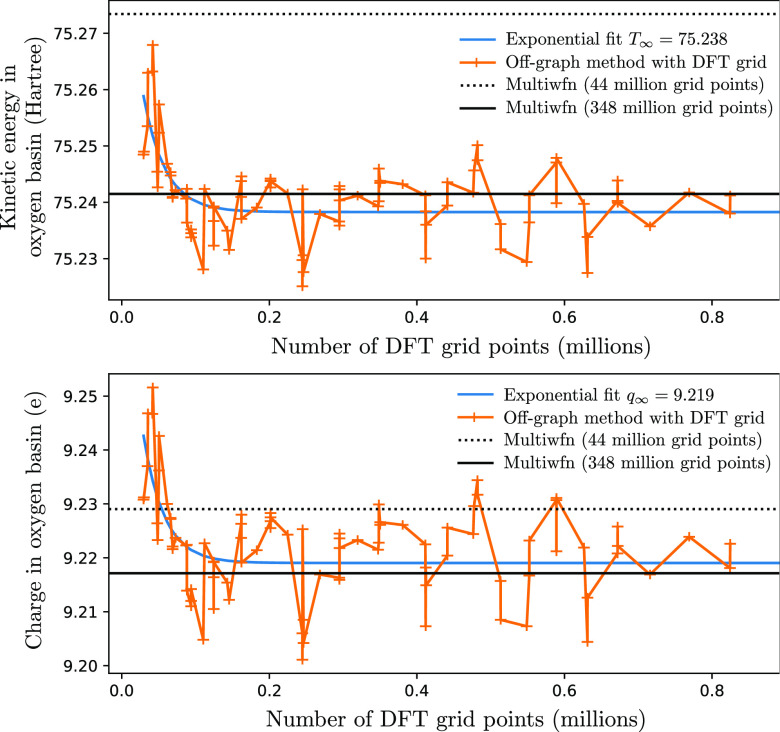
Detail of basin-integrated quantities for the water molecule using the off-graph method with DFT grids.

Thanks to the favorable properties of the DT (see Section 2.3.1), topological properties, such as the number of maxima and saddle points and so forth, converge almost instantly. Topographical properties (such as Bader charges) also converge quickly, as can be seen in Figures 17–19 where convergence is achieved for DFT grids well before 1 million grid points. Results using the uniform-grid Multiwfn package, v3.8^36^ with charge densities calculated using QChem, v5.0^35^ are also shown (the same numbers are obtained if Psi4 v1.6.1^42^ is used in place of QChem). Such uniform-grid methods routinely use tens of millions of grid points^43^—we quote results obtained using Multiwfn’s “Lunatic quality grid” (of order 10 million points) and using an even larger custom grid (of order 100 million points, obtained by specifying a custom spacing), which we denote as *extra-lunatic*, which was necessary to achieve convergence in all cases.

DFT grids converge particularly quickly as they are designed for rapid convergence of integral quantities, but even the uniform grids shown in Figure 17 perform well thanks to their connectivity with a triangulation, rather than a simple grid (see also Figure 21). Performance on such uniform grids is particularly important in calculations involving a plane-wave basis set, where real-space properties are most naturally evaluated on a uniform grid via a fast Fourier transformation.

**Figure 20:**
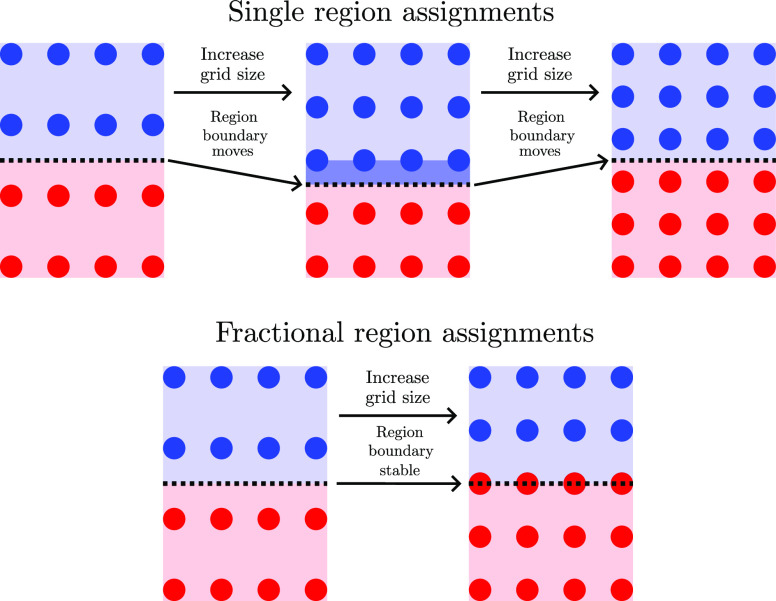
Illustration of how fractional region assignments might help to reduce convergence noise.

While the graph algorithm exhibits rapid convergence with grid size, the convergence is quite noisy—as can be seen in detail in Figure 19. However, this noise is on the order of (or smaller than, in the case of the kinetic energy) the difference between the *lunatic* and *extra-lunatic* Multiwfn grids using 4 orders of magnitude fewer grid points than the latter. One potential method to reduce this convergence noise would be to assign fractional basin weights from each grid point to each basin, as illustrated in Figure 20. Given that we already construct the DT, which allows for rapid calculation of the Voronoi tiling, the weighting method proposed in ref (26) would be particularly suitable.

#### 2.8.2 Grid Bias

**Figure 21:**
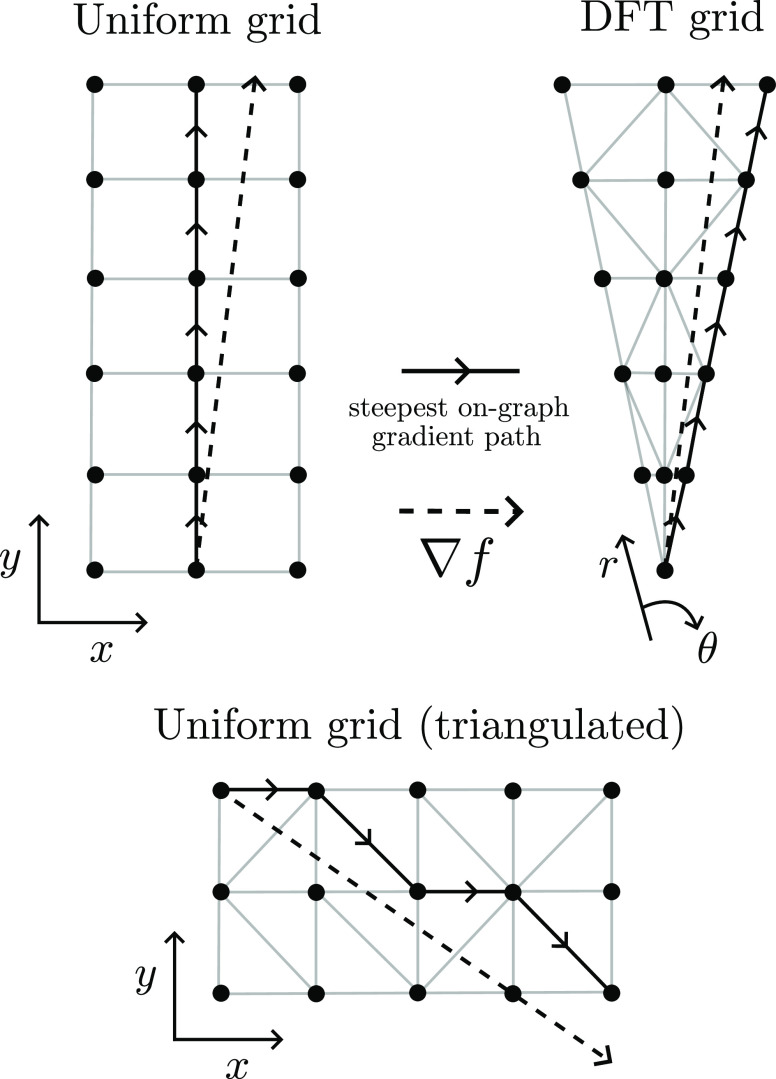
Divergence of steepest on-graph paths from the true gradient ∇*f* for different grids. At each step of the steepest-ascent path, the gradient is followed as closely as possible on the graph, but small errors at each step accumulate and the paths eventually diverge. Note that the diagonal moves introduced into a uniform grid via triangulation help (more so in 3D), but do not remove the problem. The easiest way to see that this problem is scale-independent is by considering the upper-left uniform grid case—the steepest ascent path will always be “upward” (the horizontal moves will never be taken), regardless of the grid spacing.

As noted in ref (43) for uniform grids, topographical properties (such as Bader charges) can be affected by a *grid bias*, whereby a systematic error arises due to geometrical properties of the arrangement of grid points. This error is due to steepest-ascent paths on the graph diverging from the true gradient path of the function, and, remarkably, persists even in the infinite-grid-density limit. In particular, gradient paths which are nearly, but not quite, aligned with move directions on the graph will lead to gradually diverging steepest-ascent paths, as can be seen in Figure 21, leading to distorted region boundaries.

**Figure 22:**
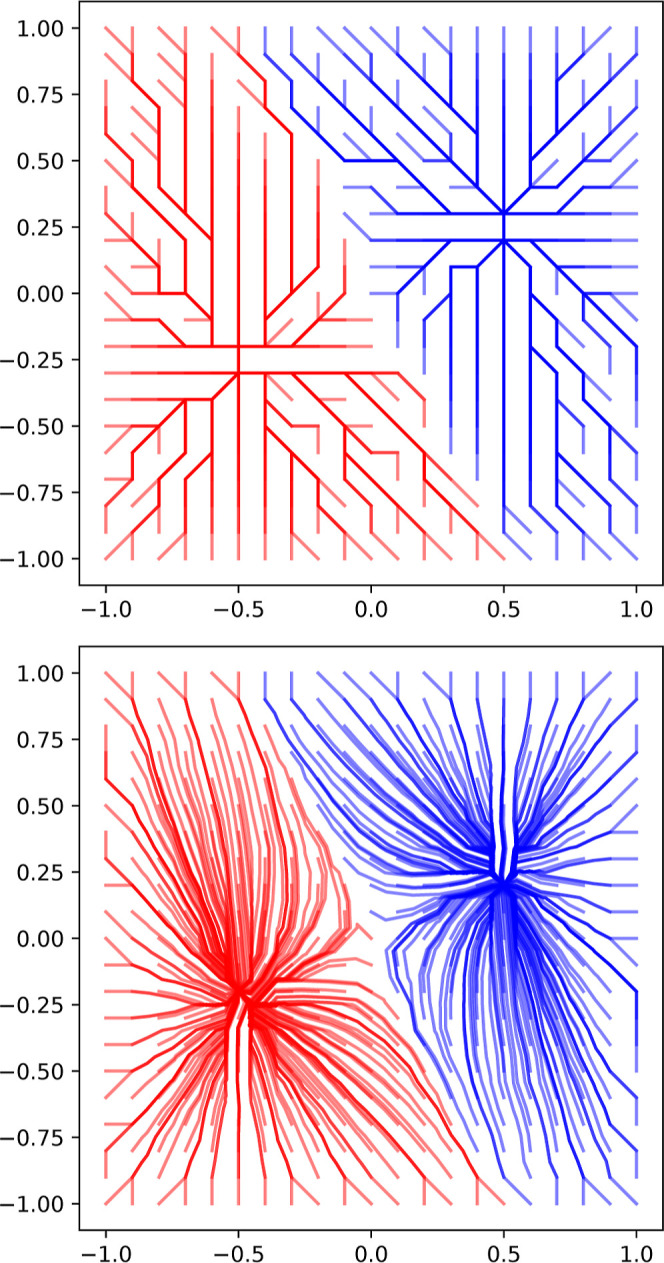
Gradient paths generated using both the on-graph method (top) and the off-graph method (bottom), described in Section 2.8.2, for a function with two maxima on a uniform 2D grid.

Reference (43) provides a solution to the grid bias problem by allowing the trajectory of ascent paths to go “off-grid”. For the graphs employed in this work, an analogous “off-graph” method can be straightforwardly implemented by allowing our ascent path to move freely in , following the nearest neighbour gradient *g*_NN_(*x*) given by
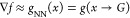
21where *g*(*x*) is the finite-difference gradient given by eq 6. The nearest-neighbor lookup *x* → *G* (see eq 11) can be implemented efficiently as a KD-tree.^44^ An example of the resulting off-graph gradient paths is shown in Figure 22 (bottom). In Figure 17, it is clear that this technique corrects the grid bias for a DFT grid so that it agrees with the (also corrected) uniform result. The uniform grid shows significantly less grid bias in Figure 17 due to the inclusion of diagonal moves by the DT (the DFT grid also has such diagonal moves, but they are less helpful as a significant radial bias remains). These diagonal moves were not present in previous uniform-grid-based approaches,^43^ which therefore exhibited significantly larger grid bias than the present method. We note that the methods developed by Rodríguez et al.^19−21^ are inherently off-grid and so do not suffer from grid-bias at the cost of requiring it to be possible to evaluate *f* and ∇*f* freely. In contrast, eq 21 requires no evaluations of *f* beyond those given as input (eq 1) at the expense of a KD-tree lookup.

#### 2.8.3 Scaling

**Figure 23:**
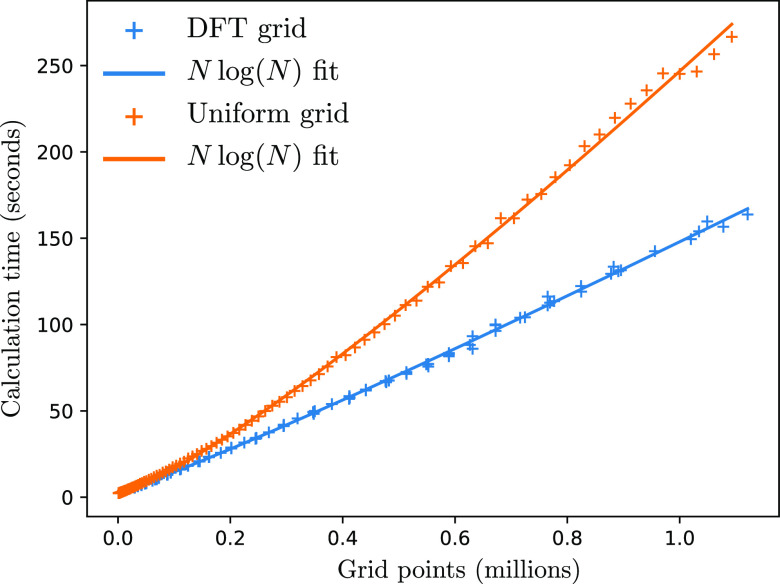
Time scaling for Bader analysis of a water molecule as a function of grid size for both a uniform and a DFT grid.

The rate-limiting step in performing topological analysis via a graph over grid points is the construction of the DT which scales as *O*[*N* log(*N*)] in the number of grid points *N*.^45^ This scaling is reflected in our calculation times (see Figure 23). Note that we use a largely unoptimized python code, so the absolute time shown on the *y* axis of Figure 23 could be improved relatively easily if desired, but the scaling will remain *O*(*N* log(*N*)).

## 3 Summary

A method has been presented to extract topographical and topological properties of a function defined on an arbitrary set of points in space, strictly via post-processing with no additional function evaluations. By connecting the points with a neighborhood graph, well-defined and robust algorithms were developed that allow for identification of local and global maxima (both point-like and spatially extended), saddle points, critical paths (and their critical points), and basins of attraction. By simple transformations of the function, one can also identify local and global minima, isosurfaces, and stagnation graphs. Applications of the analysis were demonstrated for a few problems in quantum chemistry including Bader charge and bond analysis, identification of valence shells and their charge concentration, location of lone pairs via the ELF or the Laplacian of the electron density, and identification of stagnation graphs of magnetically induced currents. All of these investigations were carried out directly on a real-space integration grid used in DFT calculations, allowing the results to be easily, efficiently, and directly incorporated into DFT calculations. The analysis was found to scale as *O*[*N* log(*N*)], where *N* is the number of grid points and quantities of interest were found to converge rapidly with *N*, requiring orders of magnitude fewer grid points than uniform-grid methods. Topographical results calculated using such DFT grids were found to exhibit a significant “grid bias” when the algorithm was constrained to stay on the graph. The source of this bias was analyzed and found to be removed by allowing off-graph moves.

## Appendix A

### Notation Primer

What follows is a primer on some mathematical constructs used in the paper, with the hope that this will make it accessible for a wider readership.

#### 4.1 Set Notation

We make frequent use of set notation of the following form

A1

where the colon can be read as “such that”. We also employ common symbols for the set of real numbers  and natural numbers . For example, we could write the set of real numbers between 0 and 1 (inclusive) as

A2

Which one could read as “The set of *x* in the real numbers such that *x* is greater than or equal to 0 and less than or equal to 1”. Here, the symbol ∈, read as “in”, indicates that *x* is a member of the set of real numbers . Some other sets that appear in the paper include the empty set ϕ and the set  of all length-*N* vectors with real entries. We also employ several set operations, in particular ∩, ∪ , and / for the intersection, union, and subtraction of two sets, respectively, so that

A3

A shorthand for such an interval on the real numbers is *S* = [0, 2]. We also employ subscripted set operations such as

A4

minimizers over sets such as

A5

and set relations such as the subset ⊂ and superset ⊃

A6

#### 4.2 Graphs

A *graph* is a set of *nodes* that may be connected by *edges*. The edges can be either *undirected* (the connection goes both ways) or *directed* (the connection is only one-way), as shown below.

The edges of a graph may also have associated *weights* (resulting in a *weighted graph*). For example, a one-way road network might be represented as a directed graph with junctions given by nodes and roads represented by edges with weights equal to the road lengths.

The notion of a *subgraph* is also used. A graph *S* is a subgraph of *G* (*S* ⊂ *G*) if *S* can be obtained from *G* by removing edges or nodes. Certain subgraphs are of particular importance in the present work. In particular, we make reference to the *minimum spanning tree* (MST) of a weighted, undirected, and connected graph (connected here meaning that it is possible to reach any node from any other node via a path along edges). The MST is the connected subgraph that minimizes the total edge weight, and so can be expressed as

A7

where

A8

is the set of connected subgraphs of *G*.

We describe several algorithms in the context of graphs. For example, consider the *flood fill* algorithm, which visits nodes in order of increasing distance from some initial node, described step-wise as

1 Initialize: Let *x* be a node of the graph *G* and *V* = {*x*} be the set of visited nodes.
2 Search: Find the set of nodes that are accessible from nodes in *V* via the edges of *G*; *S* = ∪_*x*∈*V*_*G*(*x*), where *G*(*x*) is the set of nodes accessible along edges of *G* from *x*.
3 Identify unvisited: Of the set *S*, identify the unvisited nodes *U* = *S*/*V*. If *U* = ϕ, terminate the algorithm.
4 Expand: *V* → *V* ∪ *U*.
5 Loop: Return to step 2.

Along with some of the set theory notation introduced earlier, we have used an arrow →, which can be read as “goes to” to signify updating a set. The progression of the flood fill algorithm is illustrated for a simple graph below

The sets *V*, *S*, and *U* are shown at the end of step 3 for a given iteration. Note how the set *V* gradually *floods* the entire graph, starting from the initial point.
